# Using Wearable Passive Sensing to Predict Binge Eating in Response to Negative Affect Among Individuals With Transdiagnostic Binge Eating: Protocol for an Observational Study

**DOI:** 10.2196/47098

**Published:** 2023-07-06

**Authors:** Emily K Presseller, Elizabeth W Lampe, Fengqing Zhang, Philip A Gable, Timothy C Guetterman, Evan M Forman, Adrienne S Juarascio

**Affiliations:** 1 Department of Psychological and Brain Sciences Drexel University Philadelphia, PA United States; 2 Center for Weight, Eating, and Lifestyle Science Drexel University Philadelphia, PA United States; 3 Department of Psychological and Brain Sciences University of Delaware Newark, DE United States; 4 Department of Family Medicine University of Michigan Medical School Ann Arbor, MI United States

**Keywords:** affect, binge eating, heart rate, heart rate variability, electrodermal activity, ecological momentary assessment, wearable sensors, ecological momentary intervention

## Abstract

**Background:**

Binge eating (BE), characterized by eating a large amount of food accompanied by a sense of loss of control over eating, is a public health crisis. Negative affect is a well-established antecedent for BE. The affect regulation model of BE posits that elevated negative affect increases momentary risk for BE, as engaging in BE alleviates negative affect and reinforces the behavior. The eating disorder field’s capacity to identify moments of elevated negative affect, and thus BE risk, has exclusively relied on ecological momentary assessment (EMA). EMA involves the completion of surveys in real time on one’s smartphone to report behavioral, cognitive, and emotional symptoms throughout the day. Although EMA provides ecologically valid information, EMA surveys are often delivered only 5-6 times per day, involve self-report of affect intensity only, and are unable to assess affect-related physiological arousal. Wearable, psychophysiological sensors that measure markers of affect arousal including heart rate, heart rate variability, and electrodermal activity may augment EMA surveys to improve accurate real-time prediction of BE. These sensors can objectively and continuously measure biomarkers of nervous system arousal that coincide with affect, thus allowing them to measure affective trajectories on a continuous timescale, detect changes in negative affect before the individual is consciously aware of them, and reduce user burden to improve data completeness. However, it is unknown whether sensor features can distinguish between positive and negative affect states, given that physiological arousal may occur during both negative and positive affect states.

**Objective:**

The aims of this study are (1) to test the hypothesis that sensor features will distinguish positive and negative affect states in individuals with BE with >60% accuracy and (2) test the hypothesis that a machine learning algorithm using sensor data and EMA-reported negative affect to predict the occurrence of BE will predict BE with greater accuracy than an algorithm using EMA-reported negative affect alone.

**Methods:**

This study will recruit 30 individuals with BE who will wear Fitbit Sense 2 wristbands to passively measure heart rate and electrodermal activity and report affect and BE on EMA surveys for 4 weeks. Machine learning algorithms will be developed using sensor data to distinguish instances of high positive and high negative affect (aim 1) and to predict engagement in BE (aim 2).

**Results:**

This project will be funded from November 2022 to October 2024. Recruitment efforts will be conducted from January 2023 through March 2024. Data collection is anticipated to be completed in May 2024.

**Conclusions:**

This study is anticipated to provide new insight into the relationship between negative affect and BE by integrating wearable sensor data to measure affective arousal. The findings from this study may set the stage for future development of more effective digital ecological momentary interventions for BE.

**International Registered Report Identifier (IRRID):**

DERR1-10.2196/47098

## Introduction

Binge eating (BE), characterized by perceived overeating accompanied by a subjective sense of loss of control over eating [1], is a public health crisis associated with significant psychological and physical morbidity [2-6]. The frontline treatment for BE, cognitive behavioral therapy, fails to produce remission in 40%-70% of individuals [7,8]. There is a clear need to better understand specific risk factors for BE that could be targeted to improve outcomes.

The affect regulation model of BE posits that affect dysregulation (ie, difficulty tolerating or regulating negative emotional states) is a significant, momentary risk factor for BE, as BE is negatively reinforced by the alleviation of negative affect that it facilitates [9-11]. Although the affect regulation model of BE has been substantiated in extant literature [12-14], there remain several unanswered questions about the role of affect in BE.

Existing literature examining affect as a momentary predictor of BE has exclusively used ecological momentary assessment (EMA) [12,13,15-20]. EMA methodologies for BE typically involve completing multiple daily surveys on a smartphone to report binge episodes and contextual risk factors for BE. Although EMA allows for the examination of within-day relationships between affect and BE, these surveys are completed only every few hours, which precludes the examination of minute-to-minute changes in affect [21]. The field has already identified some inconsistent findings on the role of affect in predicting BE that have been attributed to the inability to measure proximal changes to affect before and after BE with EMA [14-16,18-20,22,23]. For example, an individual may experience rapid-onset negative affect related to an event (eg, intense anxiety or sadness upon hearing bad news) and engage in BE to quell the negative affect within the span of minutes, in contrast to the hours-long timescale on which the relationship between EMA-measured affect and BE is typically examined. Thus, it is critical to examine continuously measured affect to uncover the timescale on which negative affect increases BE risk.

Existing research using EMA to study BE has also focused on only 1 of the 2 key aspects of affect, affective valence (ie, the extent to which an affective state is pleasant or unpleasant). Measuring markers of affective arousal (ie, the degree of associated physiological activity) alongside affect valence may improve prediction of BE, as affect arousal may increase eating-related disinhibition [24] and the aversiveness of negative affect states [25].

A novel data collection method that could address the limitations of EMA is ambulatory passive sensing. Sensors are likely to augment EMA for the real time measurement of affect as they permit objective, continuous, and passive measurement of biomarkers of affective arousal. The use of sensors to measure physiological correlates of affect will allow for the observation of affect trajectories immediately before, during, and after EMA-reported binge episodes. Sensor data may also be able to detect negative affect before the individual is consciously aware of it, potentially facilitating earlier detection of risk for BE. Passive sensors also require substantially lower user engagement than EMA, which may produce higher compliance with data collection and reduce the requisite frequency of EMA.

Established biomarkers of affect that can be measured by ambulatory sensors include heart rate, heart rate variability, and electrodermal activity [26,27]. These features are measures of sympathetic nervous system arousal (eg, elevated heart rate, increased electrodermal activity) and parasympathetic nervous system inactivation (eg, decreased heart rate variability), which are hallmarks of affective arousal. A substantial number of studies have identified reliable changes in heart rate, heart rate variability, and electrodermal activity associated with negative affect [28-35], but only 2 studies have used ambulatory sensors to assess heart rate variability prior to BE [36,37]. These studies found that heart rate was elevated, and heart rate variability decreased prior to loss of control eating episodes relative to nonloss of control eating episodes, offering preliminary evidence that these variables may be biomarkers of affect that confer BE risk [36,37]. However, these studies were conducted among adolescents and retrospectively compared heart rate indices prior to loss of control and nonloss of control eating episodes, rather than examining the accuracy of using heart rate data patterns to predict engagement in BE. Using passive sensors to collect momentary, naturalistic affective data is likely to improve our understanding of the role of affect in maintaining BE and our capacity to accurately predict future BE.

One challenge to the use of passive sensors to measure affect is that physiological arousal may also be elevated during some high-arousal positive affect states in addition to negative affect states. Some previous research using sensors to measure affective arousal has identified distinct physiological features associated with negative affect such as longer duration of physiological arousal [38], greater changes in electrodermal activity and heart rate, and greater variability in interbeat intervals associated with negative affect compared to positive affect [27,35,39,40]. Furthermore, models have been developed that can distinguish negative and positive affect based on physiological parameters in healthy individuals [41,42]. Given the incredibly large and dimensional data sets yielded by sensors, these previously developed models have used machine learning, an analytic approach well-suited to predict an output (ie, negative or positive affect) based on several input variables (eg, heart rate variability and electrodermal activity). However, the capacity for these models of sensor data to distinguish negative and positive affect has not yet been established among individuals with BE. Existing research using psychophysiological sensors among individuals with BE has demonstrated elevated autonomic reactivity to psychological stress in these individuals [43-45]. However, obesity, which affects 28%-42% of individuals with BE [3], has been associated with reduced sympathetic activity [46,47]. Given these contradictory findings, it is important to establish whether sensors can distinguish negative and positive affect in this specific population. If passive sensors can distinguish between positive and negative affect states, these sensors will provide information about both the occurrence of negative affect and the intensity of physiological arousal associated with that affective state. Even if passive sensors cannot distinguish positive and negative affect, information about the intensity of affective arousal may still improve the capacity to detect risk for BE above EMA alone.

Importantly, the use of sensor technologies to improve the field’s capacity to predict risk for BE offers transformative potential to power momentary interventions that target negative affect. Improved prediction of BE could facilitate the development of more effective just-in-time adaptive interventions that promote the use of therapeutic skills (eg, emotion regulation skills) when an individual is at identified risk for BE. Accordingly, this study will use psychophysiological sensors and EMA to measure negative affect to improve the prediction of BE. The study will evaluate whether the features extracted from passive sensor data can distinguish positive and negative affect in individuals with BE (aim 1). Consistent with previous research using sensors to distinguish positive and negative affect in healthy populations, we hypothesize that sensor features will distinguish between EMA-reported negative and positive affect with ≥60% accuracy [33]. Second, the study aims to develop and compare machine learning algorithms using sensor- and EMA-measured negative affect to predict BE episodes. We hypothesize that the combined sensor- and EMA-based machine learning algorithm will be more accurate for predicting BE than the algorithm using only EMA data. Based on past findings using sensors to predict emotional eating and dietary lapses, we hypothesize that the combined sensor and EMA algorithm will classify the occurrence of BE episodes with ≥70% accuracy [48,49].

## Methods

### Study Design

This study will use an observational design to examine real-time associations between affect (measured by psychophysiological sensors and EMA) and BE.

### Participants

Participants will be 30 individuals with recurrent BE (defined as at least 12 BE episodes in the past 3 months); we will enroll participants with objective binge episodes, subjective binge episodes, or a combination of both, as both behaviors are hypothesized to be maintained via the affect regulation model. Participants will be eligible for this study if they are 18-65 years old, have a BMI of ≥18.5 kg/m^2^, own a smartphone, and are willing to wear a smartwatch and complete EMA surveys for the 4-week study protocol. Exclusion criteria include inability to speak, read, and write English fluently, current eating disorder–focused treatment, and current, severe psychopathology that would inhibit engagement in study protocols (eg, active suicidality, psychosis, and substance use disorder). As antecedents for BE episodes are similar in bulimia nervosa, binge eating disorder, and other specified feeding and eating disorder diagnoses, individuals with any of these diagnoses will be eligible for the study provided they meet all other inclusion or exclusion criteria. We will aim to enroll 15 female (50%) and 15 male participants (50%). Furthermore, given that individuals with minoritized racial and ethnic identities have been underrepresented in eating disorders research, we aim to enroll at least 15 participants (50%) belonging to minoritized racial or ethnic groups.

### Study Recruitment and Procedures

Participants will be recruited from across the United States using social media and radio advertisements and ResearchMatch. Participants will complete an initial phone screen, followed by a baseline assessment to confirm eligibility. Participants will complete the Eating Disorder Examination, a diagnostic interview, at the baseline assessment to confirm eligibility and assess BE frequency [50]. Once eligibility is confirmed, participants will be enrolled in the study and begin the data collection protocol. Participants will be oriented to the use of the smartwatch and completion of EMA surveys at the baseline assessment. Participants will then complete EMA surveys and wear the Fitbit Sense 2 smartwatch daily for 28 days. Participation will be fully remote, with assessments conducted using Drexel Health Insurance Portability and Accountability Act–compliant Zoom platform and Fitbit Sense 2 sensor watches mailed to and returned by participants via prepaid shipping labels. Informed consent will be obtained from all participants.

### Ethics Approval

Study procedures have been approved and will be overseen by Drexel University’s institutional review board (approval received September 2022; protocol number 2207009376). All procedures will be conducted in accordance with the Helsinki Declaration.

### Measures

#### Psychophysiological Sensors to Measure Affect

Fitbit Sense 2 watches are wrist-worn devices that include several psychophysiological sensors, including photoplethysmography sensor, accelerometer, galvanic skin response sensor, and infrared thermopile. The heart rate features extracted from data collected by the photoplethysmography sensor will include time domain features, including average heart rate and root mean square of successive differences between normal heart beats (a measure of average variability in heart rate), and frequency domain features, including high-frequency heart rate variability (a measure of parasympathetic activity), and low-frequency heart rate variability (a measure of sympathetic arousal). These components were selected as they have been shown to change during conditions of elevated affective arousal among individuals with BE in laboratory and ambulatory studies [36,43,45,51,52]. Electrodermal activity features to be extracted from galvanic skin response data are tonic skin conductance level (average skin conductance level) and skin conductance responses (number of spikes in skin conductance), as these indices increase during periods of elevated negative affect among individuals with EDs characterized by BE [52-54]. The Fitbit Sense 2 watches contain sensors similar to devices that have demonstrated high validity for the measurement of heart rate indices and acceptable validity for the measurement of electrodermal activity during stressors [54-56].

#### EMA-Measured Eating Behaviors and Affect

Participants will complete signal-contingent and event-contingent EMA surveys during waking hours to report affect and the occurrence of BE and non-BE episodes (see Multimedia Appendix 1 for EMA questions). Signal-contingent surveys will be sent 6 semirandom times per day via Ethica EMA software (Ethica Data) [57]. Participants will be instructed to complete event-contingent surveys whenever they engage in BE (defined as overeating accompanied by a sense of loss of control). Consistent with BE EMA literature [12,22,58], all surveys will include ratings of positive and negative affect using the Short-Form Positive and Negative Affect Schedule modified to also include guilt, joy, excitement, and satisfaction [59]. Guilt will be included, given its established relevance to risk for BE [18] and joy, excitement, and satisfaction will be included as these items have high face validity with positive emotions individuals endorse, do not overlap with already included positive and negative affect schedule items, and have been used in previous EMA research on BE [60]. These items will be rated on a 5-point scale (1=very slightly or not at all to 5=extremely) and instances where a participant rates an item greater than 1 SD above their mean level (computed from all EMA ratings across the full study period) will be considered an instance of high negative affect or positive affect.

### Statistical Analyses

#### Aim 1

To evaluate aim 1, instances of high positive and high negative affect will be identified from EMA data; instances of concurrent high negative and high positive affect (if observed) will be excluded from analysis for aim 1, as binary classification machine learning models require mutually exclusive, binary outcomes [61]. Sensor features from the 30-minute period surrounding (15 minutes before and 15 minutes after) EMA surveys with high positive or negative affect ratings will be included in the machine learning models. This time frame was selected, given prior research indicating that affective states typically last ≥30 minutes [62,63]. The 30-minute period will be split into six 5-minute moving windows, for which heart rate, heart rate variability, and electrodermal activity indices will be computed.

Supervised binary classification machine learning models will be developed using heart rate, heart rate variability, and electrodermal activity indices to distinguish instances of high negative affect from high positive affect. Lasso regression will be used to select features that distinguish instances of high positive and high negative affect. Machine learning models (eg, support vector machines, classification and regression trees, and neural networks) using the selected parameters will be developed using the first 3 weeks of data collected (training data) and will be evaluated for accuracy using the last week of data (test data) collected from all participants. The last week of data will be excluded from algorithm development to ensure that the algorithms can be evaluated on previously unseen data to select the most accurate algorithms and avoid overfitting. Both group-level and individual-level algorithms will be generated to reduce model variance and bias, thereby addressing concerns about both over- and underfitting [61]. This approach will balance the need for accurate prediction with developing a group-level algorithm that substantiates the general capacity to predict BE using negative affect. Given the greater importance of identifying all instances of negative affect-related risk for BE to power a future intervention than correctly classifying instances without negative affect, minimum model sensitivity thresholds of 70% will be prescribed. The best performing models will be selected by maximizing sensitivity while maintaining adequate (eg, >50%) specificity and the average accuracy will be computed for the best performing models.

#### Aim 1 Power

Previous studies conducted by our team using EMA to measure negative affect among individuals with BE indicate that these individuals endorse high negative affect at 22%-32% of surveys and high positive affect at 12%-34% of surveys. Previous EMA research on BE suggests overall rates of missing data of 9.7%-32.6% [12,22,58]. Conservatively estimating that approximately 20% of surveys will include high negative affect, 20% of surveys will include high positive affect, and 30% missing data, we expect at least 529 instances each of high negative and positive affect in the training data (~0.2×6 surveys per day×21 days×30 participants×0.7 compliance). Simulation studies have indicated that supervised machine learning models using highly dimensional data require ≥150 observations in each class to be powered at 0.80 to detect a small to medium effect, which 529 exceeds [64].

#### Aim 2

Two sets of supervised binary classification machine learning models will be developed to predict BE, 1 set using only EMA-reported negative affect data and the other integrating both EMA- and sensor-measured negative affect. Due to the dearth of research examining the timescale on which negative affect increases risk for BE, sensor patterns across several timescales (ie, 5-, 15-, 30-, and 60 minutes prior to the binge episode) will be included in the models, and the timescales that maximize model sensitivity will be retained. These time periods will be divided into 5-minute moving windows, for which heart rate, heart rate variability, and electrodermal activity indices will be computed. Sensor features computed for each 5-minute window within the selected timescale will be included as predictors in each machine learning algorithm examined. As in aim 1, both group- and individual-level algorithms will be generated, and the algorithms will be developed using the first 3 weeks of data (training data) and evaluated on the last week of data (test data). Sensitivity will be set a priori >70% and the best fitting model for EMA-reported negative affect alone and for the combined data will be selected by maximizing sensitivity while maintaining adequate specificity.

#### Aim 2 Power

Previous studies conducted by our team and others have identified an average of 2-6 BE episodes per week per participant, with most episodes occurring on separate days [12,22,65,66]. Conservative estimates of 3 binge episodes per week will yield approximately 189 BE episodes (~3 episodes per week×3 weeks×30 participants×0.7 compliance) and 1134 non-BE episodes (~18 episodes per week×3 weeks×30 participants×0.7 compliance) in the training data. As in aim 1, since the number of observations in each class exceeds 150, the supervised machine learning models will be sufficiently powered [64]. 95% bootstrap CIs will be constructed for each set of models (combined and EMA-only) to evaluate their accuracy; if the intervals do not overlap, this indicates that 1 model is significantly more accurate. For the bootstrap CI comparison, a power analysis conducted in G*Power (version 3.1; Heinrich-Heine-Universität) (power=0.80; α=.05) indicated that ≥77 total test observations are required to detect a medium effect. Aim 2 will therefore be fully powered, given 63 binge episodes (~3 episodes per week×1 week×30 participants×0.7 compliance) and 378 anticipated non-BE episodes (~18 episodes per week×1 week×30 participants×0.7 compliance) in the test data [67].

## Results

This study received funding from the National Institute of Mental Health (award number F31MH131262) for the period of November 2022 through October 2024. Recruitment began in January 2023 and is expected to continue through March 2024; as of April 2023, a total of 7 participants have been enrolled. We anticipate that data collection will be completed by May 2024, and all analyses will be conducted by July 2024.

## Discussion

### Principal Findings

We anticipate that findings from this study will clarify whether features extracted from passive sensor data can distinguish between positive and negative affect among individuals with BE and provide an estimate of the accuracy with which these features can distinguish these emotional states. Furthermore, the study findings will identify which features are useful for distinguishing positive and negative affect states, which we anticipate will include both heart rate and electrodermal activity features. The findings from the study will also clarify the extent to which sensor-measured negative affect improves upon EMA-measured negative affect for the accurate prediction of BE episodes and provide an estimate for the accuracy of machine learning algorithms using these data sources to predict BE.

If our hypotheses are supported, our findings will replicate previous algorithms using sensor data to distinguish positive and negative affect states in a population of individuals with BE [33]. Furthermore, this study will build upon previous research using psychophysiological sensors to measure autonomic indices of emotional arousal [28-35] and to assess autonomic activity prior to BE [36,37] among individuals with BE by using these sensors to predict ecological BE in real time.

### Study Implications and Future Directions

This study will use an innovative data collection method to detect real-time risk for BE, potentially providing a new avenue to study the role of affect in BE. This study may set the stage for a future sensor-integrated momentary intervention system, a technological innovation that could enhance the efficacy of mobile health tools. The findings from this study will be disseminated via presentation at scientific conferences and publication in a peer-reviewed journal.

### Strengths and Limitations

This study is strengthened by the use of psychophysiological sensors and EMA to measure affect and eating behavior in an ecologically valid way. The inclusion of a transdiagnostic sample and a high proportion of men and individuals of minoritized racial and ethnic identities will increase the generalizability of our findings to groups that are underrepresented in research on BE. This study is limited by the possibility of reactivity to wearing the sensors or completing EMA surveys and the potential impact of limited participant insight and social desirability on reporting affect and BE on EMA.

## Appendix

Multimedia Appendix 1 Ecological momentary assessment questions.

Multimedia Appendix 2 Peer-review report by the National Institutes of Health Center for Scientific Review Special Emphasis Panel Fellowships: Risks, Prevention, and Health Behavior.

## Abbreviations

BE: binge eating
EMA: ecological momentary assessment

## Data Availability

The data sets generated during this study will be available from the corresponding author on reasonable request.
