# Supplementary material for: Using Wearable Passive Sensing to Predict Binge Eating in Response to Negative Affect Among Individuals With Transdiagnostic Binge Eating: Protocol for an Observational Study
Source: JMIR Res Protoc. 2023 Jul 6;12:e47098. doi: 10.2196/47098 (PMC10360009; doi:10.2196/47098)
Supplement: Multimedia Appendix 1 [file resprot_v12i1e47098_app1.docx]

**Multimedia Appendix 1. Ecological momentary assessment questions.**

*Have you eaten since your last survey?*

1 = Yes 0 = No

*Meal type:*

1 = Meal 2 = Snack 3 = Binge eating episode

*If yes, what time did your eating episode begin?*

*Did you experience loss of control?*

1 = Yes 0 = No

*Right now, to what extent do you feel afraid?*

1=Very slight/not at all 2 = A little 3 =Moderately 4 = Quite a bit 5= Extremely

*Right now, to what extent do you feel excited?*

1=Very slight/not at all 2 = A little 3 =Moderately 4 = Quite a bit 5= Extremely

*Right now, to what extent do you feel upset?*

1=Very slight/not at all 2 = A little 3 =Moderately 4 = Quite a bit 5= Extremely

*Right now, to what extent do you feel determined?*

1=Very slight/not at all 2 = A little 3 =Moderately 4 = Quite a bit 5= Extremely

*Right now, to what extent do you feel joyful?*

1=Very slight/not at all 2 = A little 3 =Moderately 4 = Quite a bit 5= Extremely

*Right now, to what extent do you feel guilty?*

1=Very slight/not at all 2 = A little 3 =Moderately 4 = Quite a bit 5= Extremely

*Right now, to what extent do you feel attentive?*

1=Very slight/not at all 2 = A little 3 =Moderately 4 = Quite a bit 5= Extremely

*Right now, to what extent do you feel nervous?*

1=Very slight/not at all 2 = A little 3 =Moderately 4 = Quite a bit 5= Extremely

*Right now, to what extent do you feel alert?*

1=Very slight/not at all 2 = A little 3 =Moderately 4 = Quite a bit 5= Extremely

*Right now, to what extent do you feel inspired?*

1=Very slight/not at all 2 = A little 3 =Moderately 4 = Quite a bit 5= Extremely

*Right now, to what extent do you feel ashamed?*

1=Very slight/not at all 2 = A little 3 =Moderately 4 = Quite a bit 5= Extremely

*Right now, to what extent do you feel active?*

1=Very slight/not at all 2 = A little 3 =Moderately 4 = Quite a bit 5= Extremely

*Right now, to what extent do you feel satisfied?*

1=Very slight/not at all 2 = A little 3 =Moderately 4 = Quite a bit 5= Extremely

*Right now, to what extent do you feel hostile?*

1=Very slight/not at all 2 = A little 3 =Moderately 4 = Quite a bit 5= Extremely
