# Supplementary material for: Using Wearable Passive Sensing to Predict Binge Eating in Response to Negative Affect Among Individuals With Transdiagnostic Binge Eating: Protocol for an Observational Study
Source: JMIR Res Protoc. 2023 Jul 6;12:e47098. doi: 10.2196/47098 (PMC10360009; doi:10.2196/47098)
Supplement: Multimedia Appendix 2 [file resprot_v12i1e47098_app2.pdf]

SUMMARY STATEMENT
(Privileged Communication)

PROGRAM CONTACT:
Ashley Smith
301-496-4406
ashley.smith2@nih.gov

Release Date: **07/18/2022**
Revised Date:

Application Number: **1 F31 MH131262-01A1**
Formerly: **1F31MH131262-01**

PRESSELLER,EMILY
DREXEL UNIVERSITY
3201 Chestnut Street, Stratton Hall
Room 288
Philadelphia, PA 191044318

Review Group:	ZRG1 F16-L (20)<br>Center for Scientific Review Special Emphasis Panel<br>Fellowships: Risks, Prevention and Health Behavior
Meeting Date:	07/07/2022
Council:	OCT 2022 — *PCC:* AK-TNF
Requested Start:	09/01/2022

Project Title:	Using Wearable Passive Sensing to Predict Engagement in Binge Eating in Response to Negative Affect: A Multimethod Investigation of Predictive Utility, Feasibility, and Acceptability
	Requested: 2 Years
Sponsor:	Juarascio, Adrienne S
Department:	College of Arts and Sciences
Organization:	DREXEL UNIVERSITY
City, State:	PHILADELPHIA PENNSYLVANIA
SRG Action:	Impact Score:10 Percentile:1 +
Next Steps:	Visit https://grants.nih.gov/grants/next_steps.htm
Human Subjects:	30-Human subjects involved - Certified, no SRG concerns
Animal Subjects:	10-No live vertebrate animals involved for competing appl.
Gender:	1A-Both genders, scientifically acceptable
Minority:	1A-Minorities and non-minorities, scientifically acceptable
Age:	3A-No children included, scientifically acceptable

1F31MH131262-01A1 Presseller, Emily

RESUME AND SUMMARY OF DISCUSSION: This application requests support for training in disordered eating behaviors and research that investigates the role of negative affect in binge-eating. In discussion, the reviewers noted that the applicant responded thoughtfully to the prior reviews. Strengths of the revised submission include an accomplished applicant with relevant research experience, excellent academic and publication records, and laudatory letters of recommendation. The mentoring team also is excellent and has the needed experience and expertise to guide the applicant to successful completion of the research and training activities. The research project focuses on the development of machine learning algorithms using sensor-collected physiological arousal data and ecological momentary assessment affective data from individuals who binge eat with the goal of predicting binge-eating episodes. Strengths of the project include the emphasis on inclusion of both binge-eating individuals and clinicians, the incorporation of mixed-methods with a clearly-described quantitative and qualitative data analytic plan, the emphasis on feasibility and acceptability, and the suitability of the collected data to inform a momentary intervention. Minor concerns were raised regarding the large number of items to be used in the assessments which could create burden. The training plan includes activities and experiences to broaden the applicant's skills and expertise. Overall, application strengths of a promising applicant, a competent and committed mentoring team, and research and training activities that support the applicant's trajectory to independence outweighed concerns; the application is outstanding.

DESCRIPTION (provided by applicant): Binge eating, characterized by eating a large amount of food in a short period of time accompanied by a sense of loss of control over eating, is a public health crisis. Negative affect is a well-established antecedent for binge eating. The affect regulation model of binge eating posits that elevated negative affect increases momentary risk for binge eating, as engaging in binge eating alleviates negative affect and reinforces the behavior. The field's existing capacity to identify moments of elevated negative affect, and thus risk for binge eating, has largely relied on ecological momentary assessment (EMA). EMA involves the completion of surveys in real time on one's smartphone to report behavioral, cognitive, and emotional symptoms throughout the day. Although EMA provides ecologically valid information about daily experiences, EMA surveys are often delivered only 5-6 times per day, involve self-report of affect intensity, and are unable to assess physiological arousal that accompanies affect. Wearable, psychophysiological sensors that measure markers of affect arousal including heart rate, heart rate variability, and electrodermal activity, may augment EMA surveys to improve our capacity to accurately detect risk for binge eating in real time. These sensors can objectively, continuous, and passively measure biomarkers of nervous system arousal that coincide with affect, thus allowing them to measure affective trajectories on a continuous timescale, detect changes in negative affect before the individual is consciously aware of them, and reduce user burden to improve data completeness. Despite their potential to improve the field's capacity to detect risk for binge eating, the feasibility and acceptability of these sensors among individuals with binge eating has not yet been established. Additionally, it is unknown whether features extracted from these sensors can adequately distinguish between positive and negative affect states, given that physiological arousal may occur during both negative and positive affect states. The aims of the present study are: 1) test the hypothesis that sensor features will distinguish positive and negative affect states in individuals with binge eating with > 60% accuracy; 2) test the hypothesis that a machine learning algorithm using sensor data and EMA-reported negative affect data to predict the occurrence of binge eating episodes will predict binge eating with greater accuracy than an algorithm using EMA-reported negative affect alone; 3) use a mixed methods approach to evaluate acceptability and feasibility of wearable sensors among individuals with binge eating. To do so, the present study will recruit 30 individuals with clinically-significant binge eating who will wear Empatica E4 wristbands to passively measure heart rate and electrodermal activity and report affect and binge eating on EMA

surveys for four weeks. Participants with binge eating (N = 30) and community eating disorder clinicians (N = 10) will also complete self-report measures and focus groups to assess the feasibility, acceptability, and user preferences regarding the use of sensors to power improved momentary interventions for binge eating.

PUBLIC HEALTH RELEVANCE: Rising negative affect is a well-established momentary maintenance factor for binge eating, yet limitations inherent to ecological momentary assessment hinder the field's capacity to detect risk for binge eating in response to negative affectivity. This study will use passive psychophysiological sensors to evaluate the momentary association between negative affect and binge eating with the goals of 1) improving upon EMA to accurately predict binge eating in real time and 2) quantitatively and qualitatively evaluating the feasibility and acceptability of wearable psychophysiological sensors among individuals with binge eating. The project will set the stage for future research to develop more effective just-in-time adaptive interventions for binge eating and improve treatment outcomes and access in this population.

CRITIQUE 1

Fellowship Applicant: 1
Sponsors, Collaborators, and Consultants: 2
Research Training Plan: 2
Training Potential: 1
Institutional Environment & Commitment to Training: 1

Overall Impact/Merit: This is a revised F31 pre-doctoral fellowship application designed to provide the applicant with training as she works towards an independent research career where she hops to develop and evaluate technology-based interventions for eating disorders. The applicant is a rising 3rd year Ph.D. student at Drexel in Clinical Psychology, where she previously served as a research coordinator. She has 15 peer-reviewed publications (5 first) in strong journals, and has numerous conference presentations. Her past research training is very strong, and her productivity suggests she is on a path to an independent research career. The proposed research study will collect sensor and self-reported EMA data from 30 adults who binge eat, and use the data to first develop, and then test, an algorithm using machine learning to identify times when binge eating may occur based on passive affect measurement. She will also receive qualitative research training and conduct focus groups to assess acceptability of using sensors for ED treatment with adults who binge eat and clinicians. Training goals include developing content expertise in theories of affect, role of affect in binge eating, and affect measurement; expertise in statistical methods for machine learning; expertise and training in qualitative and mixed methods research. These goals are consistent with the research proposed, and will fill in training gaps for the applicant. The co-sponsors and mentorship team is very strong and appropriate for this application, as is the training environment. Overall, only very minor concerns remain regarding this application related to the proposed research related to the appropriateness of the large number of NA and PA items, both for feasibility/burden and usable instances of only high NA or PA for the algorithms. However, this is a very strong applicant, project, and training plan, with a strong potential for propelling the applicant to an independent research career.

1. Fellowship Applicant:

Strengths

- Applicant is rising 3rd year clinical Ph.D. student at Drexel, with a strong academic record.

- She has a very strong research background, as an undergrad at Johns Hopkins and a research coordinator at Drexel.
- She has strong publishing record given her career stage (15 pubs, 5 first author in appropriate journals), on topic areas relevant to her growing program of research.
- She describes her career goal as having a research career, and her past productivity suggests she can develop into an independent researcher.
- Applicant has very strong letters of recommendation.

Weaknesses

- None noted by reviewer.

2. Sponsors, Collaborators, and Consultants:

Strengths

- The sponsor, Dr. Juarascio, is an assistant professor of psychology at Drexel. She has a very strong grant and publication record in eating disorder research and technology-based interventions for eating disorders, making her an appropriate mentor for this applicant.
- Dr. Forman is a co-primary sponsor with Dr. Juarascio. He is a professor of psychology at Drexel and director of the WELL Center. He has a strong publication, grant, and mentoring track record to support the applicant.
- Co-sponsor, Dr. Zhang, is an associate professor of psychology at Drexel and will provide statistical training and support for the applicant. Her expertise is in senor data analysis and machine learning, so will support the applicant in her training in these areas.
- Dr. Gable was added as a co-sponsor on the application, and he will mentor the applicant in passive sensing of affect. He is an associate professor of psychology at U of Delaware.
- Dr. Guetterman was also added as a co-sponsor and has expertise in mixed methods research; he will provide guidance on the qualitative component of the proposed training and study. He is an assistant professor at U of Michigan.
- The diverse mentoring team has expertise from mentors covering all major areas of the training plan. There is a good mix of more junior and senior mentors, which is a strength to this team.
- The revised application clarifies that the primary sponsor will cover research-related expenses for the proposed project, and training activities will be covered by Drexel and through the F31 institutional allowances.

Weaknesses

- None noted by reviewer.

3. Research Training Plan:

Strengths

- Research training centers around a study that uses a mixed methods approach, and has appropriate training and mentors for all components.
- The proposed study will develop machine learning algorithms for adults who binge eat, based on data collected from wristband sensors and EMA assessing PA and NA.

- Focus groups with all participants (adults who binge eat) and clinicians treating eating disorders will provide qualitative data about the use of the mobile tech to eventually inform a momentary intervention for binge eating.
- This work represents a line of research that is independent, but related to, the sponsor and mentorship team.

Weaknesses

- Consider shortening the number of PANAS items used from 21 (10 PA and 10 NA items in the PANAS-S, plus guilt). In addition to higher burden, with this many items there could be many instances where people score >1SD about their mean for a single item, and per the revised proposal, these instances of both a “high” PA and “high” NA item will not be used for the algorithm development.
- Providing references or supporting data for the estimate that 20% of surveys will have high NA and 20% will have high PA in this sample would increase confidence in the expected number of observations in the training data. Particularly given that observations where both high PA and NA are present will not be used.

4. Training Potential:

Strengths

- Training includes individual meetings with all sponsors, courses, and workshops that are appropriate for the applicant’s training goals.
- The quantity of trainings is more appropriate in the revised application to allow sufficient time for other research activities.
- Training proposed is appropriate for expanding the applicant’s training in statistical methods and content areas relevant to her career goals.
- This is an ambitious but appropriate training plan that will set the applicant up well for a future research career.

Weaknesses

- None noted by reviewer.

5. Institutional Environment & Commitment to Training:

Strengths

- Drexel and the WELL Center have a strong environment to support this applicant and proposal.

Weaknesses

- None noted by reviewer.

Protections for Human Subjects:

Acceptable Risks and Adequate Protections

- Acceptable plan for protections.

Data and Safety Monitoring Plan (Applicable for Clinical Trials Only):

Not Applicable (No Clinical Trials)

Inclusion Plans:

- Sex/Gender: Distribution justified scientifically
- Race/Ethnicity: Distribution justified scientifically
- For NIH-Defined Phase III trials, Plans for valid design and analysis: Scientifically acceptable
- Inclusion/Exclusion Based on Age: Distribution justified scientifically
- Age 18-65, justified.
- Half male, half female with binge eating and 70/30 female/male for clinicians, justified.
- Racial minority individuals will be appropriately oversampled.

Vertebrate Animals:
Not Applicable (No Vertebrate Animals)

Biohazards:
Not Applicable (No Biohazards)

Resubmission:

- Applicant was very responsive to comments from first review.
- Revised research strategy to adjust weaknesses regarding measurement.
- Revised training plan to make it more feasible and appropriate for fellowship.

Training in the Responsible Conduct of Research:
Acceptable
Comments on Format (Required):

- Individual meetings, courses, workshops, and online trainings.

Comments on Subject Matter (Required):

- All relevant topics covered in various trainings.

Comments on Faculty Participation (Required):

- Plan for how all mentors will contribute to training.

Comments on Duration (Required):

- Varies appropriately.

Comments on Frequency (Required):

- Varies appropriately.

Resource Sharing Plans:
Not Applicable (No Relevant Resources)

Budget and Period of Support:

Recommend as Requested

CRITIQUE 2

Fellowship Applicant: 1
Sponsors, Collaborators, and Consultants: 1
Research Training Plan: 1
Training Potential: 1
Institutional Environment & Commitment to Training: 1

Overall Impact/Merit: This is a well-written application from a very strong candidate with an excellent academic record and outstanding scholarly productivity. The candidate is supported by a strong mentorship team with expertise and experience in the identified areas of need for the candidate. The team also conveys an understanding of the applicant's needs and a commitment to meeting them. The proposed training plan includes mentoring in theoretical understanding of negative affect and measurement of physiological markers of negative affect, development and evaluation of machine learning algorithms to predict engagement in binge eating, and the application of mixed methods research approaches. These skills will be developed through individualized mentorship meetings, advanced coursework, and professional development activities as the applicant implements her proposed research on whether or not wearable, psychophysiological sensors that measure markers of affect arousal and electrodermal activity may augment EMA surveys to improve capacity to accurately detect risk for binge eating in real time. The research project has a strong theoretical base and rigorous methods that should result in publishable manuscripts. Given these many strengths and no identified weaknesses, the fellowship is highly likely to enhance the applicant's potential for an independent research career in technology-based interventions for eating disorders.

1. Fellowship Applicant:

Strengths

- The applicant has an excellent academic record. She also has experience in development and execution of study protocols, recruitment and enrollment of participants, data collection, management, and analysis, lab management, and project coordination.
- The applicant has a solid track record of pursuing independent research from undergraduate through graduate school. She has received three small grants to pursue her research. She has also received five awards for her work.
- The applicant has a very strong record of scholarly productivity with ten peer-reviewed papers (five as first author), six more papers under review, and 23 conference presentations (nine as first author).
- Letters of reference speak to the applicant's motivation, talent, high-level research skills, commitment to independent research, communication skills, leadership skills, problem solving ability, scholarly productivity, and notable academic track record.

Weaknesses

- None noted by reviewer.

2. Sponsors, Collaborators, and Consultants:

Strengths

- The primary sponsor (Juarascio) has extensive experience and expertise in use of technology to augment behavioral treatments for eating disorders. She has had many federally-funded projects in this area, an extensive record of recent and relevant scholarly productivity, and experience mentoring successful fellows.
- The primary co-sponsor (Forman) has experience in using technology-based treatments for binge eating as well as an extensive scholarly productivity record, experience as PI on federal grants, and a track record of successful mentoring.
- Co-sponsor Zhang provides needed mentorship in machine learning. Co-sponsor Gable will provide mentorship in analyzing psychophysiological data. Co-sponsor Guetterman will provide mentorship in mixed-methods research methodology. All 3 co-sponsors have a strong record of scholarly productivity as well as experience mentoring.
- There is clear evidence of a match between the applicant's interests and skill development needs and the sponsors' expertise and experience.
- Letters of commitment are included that convey an understanding of the applicant's training needs and a commitment to mentor her in these areas.
- There is evidence that the primary co-sponsor will provide funding via overhead research funds to support the applicant's research project.

Weaknesses

- None noted by reviewer.

3. Research Training Plan:

Strengths

- The proposed research project is grounded in a strong theoretical base and employs rigorous scientific methods to address three specific aims that are appropriate for addressing the overarching research question of whether or not wearable, psychophysiological sensors that measure markers of affect arousal and electrodermal activity may augment EMA surveys to improve capacity to accurately detect risk for binge eating in real time.
- The applicant has included a detailed analysis plan for both quantitative and qualitative data as well as power analyses for both.
- The inclusion of feasibility and acceptability testing with participants is a thoughtful addition to the research methods.
- The timeframe appears feasible for accomplishing the proposed research tasks.
- The applicant has included a discussion of potential research challenges and potential solutions.
- Results from this research are likely publishable as they fill a gap in the current literature. The training plan also includes the development of 3 manuscripts based on the proposed research.

Weaknesses

- None noted by reviewer.

4. Training Potential:

Strengths

- The proposed training plan will provide the applicant with needed training and mentoring in theoretical understanding of negative affect and measurement of physiological markers of negative affect, development and evaluation of machine learning algorithms to predict engagement in binge eating, and the application of mixed methods research approaches.
- The training plan includes individualized mentorship meetings, advanced coursework, and professional development activities such as seminar and conference attendance. There are also clear plans for manuscript writing, submission of findings to national conferences, and grant development.
- The described training plan builds on the applicant's existing strengths in research skills, while addressing specific gaps needed for independent research developing and evaluating technology-based interventions for eating disorders.

Weaknesses

- None noted by reviewer.

5. Institutional Environment & Commitment to Training:

Strengths

- Both Drexel University and the WELL Center provide strong supportive environments for the proposed research. The applicant will have access to significant opportunities for skill development and mentoring.
- The applicant will have access to participants for recruitment through WELL Center resources.
- The applicant will have access to needed wearable sensors for conducting the study.

Weaknesses

- None noted by reviewer.

Protections for Human Subjects:

Acceptable Risks and Adequate Protections

- The plan for participant protections is adequate given the identified risks.

Data and Safety Monitoring Plan (Applicable for Clinical Trials Only):

Acceptable

- o While not required, the applicant has included a DSMP to address potential issues of eating disorder should they arise.

Inclusion Plans:

- Sex/Gender: Distribution justified scientifically
- Race/Ethnicity: Distribution justified scientifically
- For NIH-Defined Phase III trials, Plans for valid design and analysis: Not applicable
- Inclusion/Exclusion Based on Age: Distribution justified scientifically
- The research will include males and females, minorities and non-minorities, and adults. All of these are scientifically justified.

Vertebrate Animals:
Not Applicable (No Vertebrate Animals)

Biohazards:
Not Applicable (No Biohazards)

Resubmission:

- The resubmitted proposal is responsive to the previous critiques.

Training in the Responsible Conduct of Research:
Acceptable

Comments on Format (Required):

- Includes in person discussion with mentors, guided readings, weekly seminars, and annual workshops.

Comments on Subject Matter (Required):

- Subject matter includes common issues in research ethics, including data management, data fabrication and falsification, plagiarism, authorship and publication, mentorship practices, conflicts of interest, use of human participants in research, equitable recruitment, informed consent, and professional conduct.

Comments on Faculty Participation (Required):

- Mentorship meetings with faculty.

Comments on Duration (Required):

- Weekly one hour mentorship meetings; Weekly seminars and annual workshops vary.

Comments on Frequency (Required):

- Weekly mentorship meetings and seminars; Annual workshops.

Resource Sharing Plans:
Not Applicable (No Relevant Resources)

Budget and Period of Support:
Recommend as Requested

CRITIQUE 3

Fellowship Applicant: 1
Sponsors, Collaborators, and Consultants: 1
Research Training Plan: 1
Training Potential: 2
Institutional Environment & Commitment to Training: 1

Overall Impact/Merit: This revised predoctoral (F31) application is from a second year grad student in clinical psychology, who proposes to identify episodes of elevated negative affect in adolescents which are considered to indicate likelihood of binge eating. Excellent applicant, highly productive scholar already at an early state of her career (10 publications, 23 presentations) who is already performing at a level comparable to that of a mature scholar. Excellent team of sponsors and consultants. Applicant has adequately responded to previous reviews with appropriate revision of the application. Proposed study and training plan are appropriate for a proposed 2 year support period. An interesting project with high chance of success.

Protections for Human Subjects:

Acceptable Risks and Adequate Protections

- No concerns.

Data and Safety Monitoring Plan (Applicable for Clinical Trials Only):

Inclusion Plans:

- Sex/Gender: Distribution justified scientifically
- Race/Ethnicity: Distribution justified scientifically
- For NIH-Defined Phase III trials, Plans for valid design and analysis:
- Inclusion/Exclusion Based on Age: Distribution justified scientifically
- No concerns.

Training in the Responsible Conduct of Research:

Comments on Format (Required):

Comments on Subject Matter (Required):

Comments on Faculty Participation (Required):

Comments on Duration (Required):

Comments on Frequency (Required):

Resource Sharing Plans:

Budget and Period of Support:

Recommend as Requested

THE FOLLOWING SECTIONS WERE PREPARED BY THE SCIENTIFIC REVIEW OFFICER TO SUMMARIZE THE OUTCOME OF DISCUSSIONS OF THE REVIEW COMMITTEE, OR REVIEWERS' WRITTEN CRITIQUES, ON THE FOLLOWING ISSUES:

PROTECTION OF HUMAN SUBJECTS: ACCEPTABLE

INCLUSION OF WOMEN PLAN: ACCEPTABLE

INCLUSION OF MINORITIES PLAN: ACCEPTABLE

INCLUSION ACROSS THE LIFESPAN: ACCEPTABLE

COMMITTEE BUDGET RECOMMENDATIONS: The budget was recommended as requested.

Footnotes for 1 F31 MH131262-01A1; PI Name: Presseller, Emily Kelley

+ Derived from the range of percentile values calculated for the study section that reviewed this application.

NIH has modified its policy regarding the receipt of resubmissions (amended applications).See Guide Notice NOT-OD-18-197 at https://grants.nih.gov/grants/guide/notice-files/NOT-OD-18-197.html. The impact/priority score is calculated after discussion of an application by averaging the overall scores (1-9) given by all voting reviewers on the committee and multiplying by 10. The criterion scores are submitted prior to the meeting by the individual reviewers assigned to an application, and are not discussed specifically at the review meeting or calculated into the overall impact score. Some applications also receive a percentile ranking. For details on the review process, see http://grants.nih.gov/grants/peer_review_process.htm#scoring.

MEETING ROSTER

Center for Scientific Review Special Emphasis Panel
CENTER FOR SCIENTIFIC REVIEW
Fellowships: Risks, Prevention and Health Behavior

ZRG1 F16-L (20)
07/07/2022 - 07/08/2022

Notice of NIH Policy to All Applicants: Meeting rosters are provided for information purposes only. Applicant investigators and institutional officials must not communicate directly with study section members about an application before or after the review. Failure to observe this policy will create a serious breach of integrity in the peer review process, and may lead to actions outlined in NOT-OD-22-044 at https://grants.nih.gov/grants/guide/notice-files/NOT-OD-22-044.html, including removal of the application from immediate review.

CHAIRPERSON(S)

TERCYAK, KENNETH, PHD
PROFESSOR AND PROGRAM LEADER
CANCER PREVENTION AND CONTROL PROGRAM
GEORGETOWN LOMBARDI COMPREHENSIVE CANCER CENTER
GEORGETOWN UNIVERSITY
WASHINGTON, DC 20007

MEMBERS

ARIGO, DANIELLE R, PHD
ASSOCIATE PROFESSOR
ROWAN SCHOOL OF OSTEOPATHIC MEDICINE
ROWAN UNIVERSITY
GLASSBORO, NJ 08028

ASLIBEKYAN, STELLA, PHD
ASSOCIATE PROFESSOR
DEPARTMENT OF EPIDEMIOLOGY
SCHOOL OF PUBLIC HEALTH
UNIVERSITY OF ALABAMA - BIRMINGHAM
BIRMINGHAM, AL 35294

BEVERSDORF, DAVID QUENTIN, MD
PROFESSOR
RADIOLOGY, NEUROLOGY, PSYCHOLOGY
THOMPSON CENTER
UNIVERSITY OF MISSOURI
COLUMBIA, MO 65212

BINGENHEIMER, JEFFREY B, PHD
ASSOCIATE PROFESSOR
DEPARTMENT OF PREVENTION AND COMMUNITY HEALTH
MILKEN INSTITUTE SCHOOL OF PUBLIC HEALTH
GEORGE WASHINGTON UNIVERSITY
WASHINGTON DC, DC 20052

BROWN, RONALD TERRY, PHD
PROFESSOR AND DEAN
INTEGRATED HEALTH SCIENCES
UNIVERSITY OF NEVADA, LAS VEGAS
LAS VEGAS, NV 89154

CARTER-HARRIS, LISA, PHD
ASSOCIATE PROFESSOR
DEPARTMENT OF PSYCHIATRY & BEHAVIORAL SCIENCES
MEMORIAL SLOAN KETTERING CANCER CENTER
NEW YORK, NY 10022

CHANG, MEI-WEI, PHD
ASSOCIATE PROFESSOR
COLLEGE OF NURSING
THE OHIO STATE UNIVERSITY
COLUMBUS, OH 43210

COHEN, JULIANA FW, SCD
ASSOCIATE PROFESSOR
DEPARTMENT OF PUBLIC HEALTH AND NUTRITION
SCHOOL OF HEALTH SCIENCES
MERRIMACK COLLEGE
NORTH ANDOVER, MA 01845

COLWELL, GREGORY BRIAN, PHD
PROFESSOR
HEALTH PROMOTION AND COMMUNITY HEALTH SCIENCES
SCHOOL OF PUBLIC HEALTH
TEXAS A&M UNIVERSITY
COLLEGE STATION, TX 77843

COSTELLO, TRACY JENNIFER, PHD
DIRECTOR, POSTDOCTORAL AFFAIRS
DEPARTMENT OF BIOSTATISTICS
MOFFITT CANCER CENTER INTERNAL MEDICINE
UNIVERSITY OF SOUTH FLORIDA
TAMPA, FL 33612

DAVIS, ELIZABETH LENORE, PHD
ASSOCIATE PROFESSOR
DEPARTMENT OF PSYCHOLOGY
UNIVERSITY OF CALIFORNIA - RIVERSIDE
RIVERSIDE, CA 92521

DEW, MARY AMANDA, PHD
PROFESSOR AND PROGRAM DIRECTOR
DEPARTMENT OF PSYCHIATRY
UNIVERSITY OF PITTSBURGH
PITTSBURGH, PA 15213

DVORAK, ROBERT DANIEL, PHD
PROFESSOR
DEPARTMENT OF PSYCHOLOGY
COLLEGE OF SCIENCES
THE UNIVERSITY OF CENTRAL FLORIDA
ORLANDO, FL 32816

FOSTER, KENNETH R, PHD
PROFESSOR
DEPARTMENT OF BIOENGINEERING
SCHOOL OF ENGINEERING AND APPLIED SCIENCE
UNIVERSITY OF PENNSYLVANIA
PHILADELPHIA, PA 19104

FOTI, DANIEL JUSTIN, PHD
ASSOCIATE PROFESSOR
DEPARTMENT OF PSYCHOLOGICAL SCIENCES
PURDUE UNIVERSITY
WEST LAFAYETTE, IN 47907

FOX, AARON D, MD
ASSOCIATE PROFESSOR
DEPARTMENT OF MEDICINE
MONTEFIORE MEDICAL CENTER
ALBERT EINSTEIN COLLEGE OF MEDICINE
BRONX, NY 10467

FREDIANI, JENNIFER KOREN, PHD
ASSOCIATE PROFESSOR
NELL HODGSON WOODRUFF SCHOOL OF NURSING
EMORY UNIVERSITY
ATLANTA, GA 30322

HERON, KRISTIN E, PHD
ASSOCIATE PROFESSOR
DEPARTMENT OF PSYCHOLOGY
OLD DOMINION UNIVERSITY
NORFOLK, VA 23529

JUAREZ, PAUL D, PHD
PROFESSOR
DEPARTMENT OF FAMILY AND COMMUNITY MEDICINE
MEHARRY MEDICAL COLLEGE
NASHVILLE, TN 37208

KOPP, LISA MICHELLE, PHD
PROFESSOR
DEPARTMENT OF HUMAN DEVELOPMENT & FAMILY STUDIES
COLLEGE OF HEALTH AND HUMAN DEVELOPMENT
THE PENNSYLVANIA STATE UNIVERSITY
UNIVERSITY PARK, PA 16802

KOUROS, CHRYSTYNA D, PHD
ASSOCIATE PROFESSOR
DEPARTMENT OF PSYCHOLOGY
SOUTHERN METHODIST UNIVERSITY
DALLAS, TX 75275

MARTIN, LAURA E, PHD
ASSOCIATE PROFESSOR
DEPARTMENT OF POPULATION HEALTH
UNIVERSITY OF KANSAS MEDICAL CENTER
KANSAS CITY, KS 66160

MCMILLEN, JANEY STURTZ, PHD
DIRECTOR OF SPONSORED PROGRAMS
SCHOOL OF NATURAL & MATHEMATICAL SCIENCES
MEREDITH COLLEGE
RALEIGH, NC 27607

MCMILLEN, ROBERT C, PHD
PROFESSOR
DEPARTMENT OF PSYCHOLOGY
SOCIAL SCIENCE RESEARCH CENTER
MISSISSIPPI STATE UNIVERSITY
MISSISSIPPI STATE, MS 39762

MCMORRIS, BARBARA J, PHD
ASSOCIATE PROFESSOR
POPULATION HEALTH AND SYSTEMS COOPERATIVE
UNIVERSITY OF MINNESOTA
MINNEAPOLIS, MN 55455

MILLER, KRISTEN ELIZABETH, DPH
SCIENTIFIC DIRECTOR
NATIONAL CENTER FOR HUMAN FACTORS IN HEALTHCARE
MEDSTAR HEALTH RESEARCH INSTITUTE
WASHINGTON, DC 20008

MOLFENTER, TODD DAVID, PHD
SCIENTIST
COLLEGE OF ENGINEERING
UNIVERSITY OF WISCONSIN - MADISON
MADISON, WI 53706

PHIPPS, AMANDA IRENE, PHD
ASSOCIATE PROFESSOR
DEPARTMENT OF EPIDEMIOLOGY
SCHOOL OF PUBLIC HEALTH
UNIVERSITY OF WASHINGTON
SEATTLE, WA 98109

RASMUSSEN, ANDREW EDWARD, PHD
ASSOCIATE PROFESSOR
DEPARTMENT OF PSYCHOLOGY
FORDHAM UNIVERSITY
BRONX, NY 10458

SEVCIK, ROSE A, PHD
PROFESSOR
DEPARTMENT OF PSYCHOLOGY
GEORGIA STATE UNIVERSITY
ATLANTA, GA 30303

SIMONSEN, SARA E, PHD
ASSOCIATE PROFESSOR
COLLEGE OF NURSING - MIDWIFERY
THE UNIVERSITY OF UTAH
SALT LAKE CITY, UT 84112

STUDTS, JAMIE L, PHD
PROFESSOR
SCHOOL OF MEDICINE
ANSCHUTZ MEDICAL CAMPUS
UNIVERSITY OF COLORADO
AURORA, CO 80045

TRAPL, ERIKA S, PHD
ASSOCIATE PROFESSOR
DPT OF POPULATION AND QUANTITATIVE HEALTH SCIENCES
SCHOOL OF MEDICINE
CASE WESTERN RESERVE UNIVERSITY
CLEVELAND, OH 44106

TUBMAN, JONATHAN G, PHD
PROFESSOR
DEPARTMENT OF PSYCHOLOGY
AMERICAN UNIVERSITY
WASHINGTON, DC 20016

VANDERFORD, NATHAN LANE, PHD
ASSOCIATE PROFESSOR
DEPARTMENT OF TOXICOLOGY AND CANCER BIOLOGY
UNIVERSITY OF KENTUCKY
LEXINGTON, KY 40536

WEINBERG, ANNA E.F., PHD
ASSOCIATE PROFESSOR
DEPARTMENT OF PSYCHOLOGY
MCGILL UNIVERSITY
MONTREAL, PQ H3A 1G1
CANADA

WEIST, MARK D, PHD
PROFESSOR
DEPARTMENT OF PSYCHOLOGY
COLLEGE OF ARTS AND SCIENCES
UNIVERSITY OF SOUTH CAROLINA
COLUMBIA, SC 29208

MAIL REVIEWER(S)

DIAZ, ESPERANZA, MD
PROFESSOR
DEPARTMENT OF PSYCHIATRY
YALE SCHOOL OF MEDICINE
NEW HAVEN, CT 06519

QUARELLS, RAKALE COLLINS, PHD
ASSOCIATE PROFESSOR
DPT OF COMMUNITY HEALTH AND PREVENTIVE MEDICINE
MOREHOUSE SCHOOL OF MEDICINE
CARDIOVASCULAR RESEARCH INSTITUTE
ATLANTA, GA 30310

SEGRE, ALBERTO MARIA, PHD
PROFESSOR AND CHAIR OF COMPUTER SCIENCE
DEPARTMENT OF COMPUTER SCIENCE
GERALD P. WEEG FACULTY SCHOLAR IN INFORMATICS
THE UNIVERSITY OF IOWA
IOWA CITY, IA 52242

SCIENTIFIC REVIEW OFFICER

FARADAY, MARTHA M, PHD
SCIENTIFIC REVIEW OFFICER
CENTER FOR SCIENTIFIC REVIEW
NATIONAL INSTITUTES OF HEALTH
BETHESDA, MD 20892

Consultants are required to absent themselves from the room during the review of any application if their presence would constitute or appear to constitute a conflict of interest.
